# Volvulus of Caecum

**Published:** 1917-07

**Authors:** 


**Volvulus of Caecum.** Farganel and Brisset, Le Progres Med., May 19, page 170, report a case developing suddenly, with crises of pain and obstruction of faeces and gas but without initial vomiting. The caecum was found at necropsy twisted and in the left hypochondrium.

